# Acceleration of TAA-Induced Liver Fibrosis by Stress Exposure Is Associated with Upregulation of Nerve Growth Factor and Glycopattern Deviations

**DOI:** 10.3390/ijms22105055

**Published:** 2021-05-11

**Authors:** Catalina Atorrasagasti, Flavia Piccioni, Sophia Borowski, Irene Tirado-González, Nancy Freitag, María José Cantero, Juan Bayo, Guillermo Mazzolini, Laura D. Alaniz, Sandra M. Blois, Mariana G. Garcia

**Affiliations:** 1 Instituto de Investigaciones en Medicina Traslacional, Facultad de Ciencias Biomédicas, CONICET, Universidad Austral, Derqui-Pilar B1629, Argentina; mcatalinaa@gmail.com (C.A.); canter.ma@yahoo.com.ar (M.J.C.); jmbayo@hotmail.com (J.B.); gmazzoli@austral.edu.ar (G.M.); 2 Laboratorio de Oncología Molecular y Nuevos Blancos Terapéuticos, Instituto de Biología y Medicina Experimental (IBYME), CONICET, Buenos Aires C1428, Argentina; flapiccioni84@gmail.com; 3 Department of Obstetrics and Fetal Medicine, University Medical Center Hamburg-Eppendorf, 20246 Hamburg, Germany; sophia.borowski@charite.de (S.B.); nancy.freitag@charite.de (N.F.); 4 Experimental and Clinical Research Center, a Cooperation between the Max Delbrück Center for Molecular Medicine in the Helmholtz Association, and the Charité—Universitätsmedizin Berlin, AG GlycoImmunology, 13125 Berlin, Germany; irene_tirado@yahoo.es; 5 Georg-Speyer-Haus, Institute for Tumor Biology and Experimental Therapy, 60596 Frankfurt, Germany; 6 Laboratorio de Microambiente Tumoral, Centro de Investigaciones Básicas y Aplicadas (CIBA), Centro de Investigaciones y Transferencia del Noroeste de la Pcia. de Bs. As. (CIT NOBA UNNOBA-CONICET), Universidad Nacional del Noroeste de la Pcia. de Bs. As., Junín B6000, Argentina; ldalaniz@comunidad.unnoba.edu.ar

**Keywords:** liver fibrosis, stress-induced fibrosis, NGF, hepatoglycocode, mouse model

## Abstract

Liver fibrosis results from many chronic injuries and may often progress to cirrhosis and hepatocellular carcinoma (HCC). In fact, up to 90% of HCC arise in a cirrhotic liver. Conversely, stress is implicated in liver damage, worsening disease outcome. Hence, stress could play a role in disrupting liver homeostasis, a concept that has not been fully explored. Here, in a murine model of TAA-induced liver fibrosis we identified nerve growth factor (NGF) to be a crucial regulator of the stress-induced fibrogenesis signaling pathway as it activates its receptor p75 neurotrophin receptor (p75NTR), increasing liver damage. Additionally, blocking the NGF decreased liver fibrosis whereas treatment with recombinant NGF accelerated the fibrotic process to a similar extent than stress challenge. We further show that the fibrogenesis induced by stress is characterized by specific changes in the hepatoglycocode (increased β1,6GlcNAc-branched complex N-glycans and decreased core 1 O-glycans expression) which are also observed in patients with advanced fibrosis compared to patients with a low level of fibrosis. Our study facilitates an understanding of stress-induced liver injury and identify NGF signaling pathway in early stages of the disease, which contributes to the established fibrogenesis.

## 1. Introduction

Liver fibrosis is a wound-healing response to acute or chronic cellular injury induced by viral hepatitis, alcohol, drugs, or metabolic diseases, among others. This process involves the production of several cytokines and chemokines like transforming growth factor β (TGFβ) and platelet-derived growth factor (PDGF), leading to the differentiation and activation of hepatic stellate cells (HSCs) to a myofibroblastic phenotype with the reorganization of cytoskeletal proteins, such as α-smooth muscle actin (α-SMA) [1]. Activated HSCs increase the synthesis of extracellular matrix components, and as a result, an excessive deposition of collagen types I and II, proteoglycans, and glycoproteins is observed in the liver parenchyma [1]. Initially, fibrosis is considered a physiological mechanism to limit the inflammatory process; however, if the wound healing process persists, it becomes pathological, leading to parenchymal replacement with scar tissue and distortion of the hepatic architecture [2]. Chronic inflammation and cellular stress response developed during liver fibrosis may explain its close relationship with hepatocarcinogenesis [3].

Stress considered as “conditions where an environmental demand exceeds the natural regulatory capacity of an organism, in particular situations that include unpredictability and uncontrollability” is known to affect the homeostasis of the nervous, immune, and endocrine systems [4]. The first evidence that liver could also be affected by stress was reported by Hirose et al., demonstrating that emotional stress decreased hepatic blood flow [5]. Later, other animal studies demonstrated histologic liver damage and elevated transaminases due to electric foot shock or restraint stress [6,7,8]. In addition, some clinical data also suggested a correlation between psychosocial stress and the worsening of liver disease [9,10,11].

The mechanisms involved in the stress response have been described for pathological conditions associated to the brain, heart, immune system, and pregnancy maintenance, among others [12,13,14]. However, few reports studied the mediators involved in stress-induced liver damage. Chida et al. demonstrated that the exposure to electric foot shock as a mode of psychophysical stress exacerbated α-galactosylceramide-induced hepatitis in mice through the elevation of endogenous glucocorticoids [7]. Another report described an increase in interleukin-1β and corticosterone in a model of repeated immobilization stress in rats, causing liver damage associated with the infiltration of mononuclear cells and necrosis in the liver parenchyma [15]. However, the mechanisms by which stress affects liver physiology remains poorly understood. Moreover, most of the data related with stress and liver damage were obtained in experimental models of acute stress. In the current society, stress is a chronic and inevitable stimulus, for that reason, a better understanding of its effect on liver diseases could help to manage the treatment of these patients.

Here, we investigate whether stress exposure imposes a deleterious effect on experimental liver fibrogenesis. We observed that stress increased liver fibrosis by the upregulation of NGF. The amelioration of fibrosis development was observed by neutralization of NGF whereas exogenous NGF treatment mimics the effect of stress on liver fibrogenesis. We further showed that NGF treatment upregulated its receptor p75NTR and induced apoptosis, especially around portal veins and fibrotic tracts. Moreover, changes in the pattern of liver glycosylation, such as increased β1,6GlcNAc-branched complex N-glycans and decreased core 1 O-glycans expression was observed after stress stimulus and NGF treatment. In support of our observations, an analysis of publicly available RNAseq data shows that N-acetylglucosaminyltransferase V (MGAT-5), the enzyme involved in branched complex N-glycans formation is also increased in patients with advanced fibrosis, suggesting that specific hepatoglycocode alterations characterize the establishment of liver fibrosis.

## 2. Results

### 2.1. Stress Exposure Accelerates TAA-Induced Fibrosis and Results in Increased Liver NGF Expression

**Figure 1:**
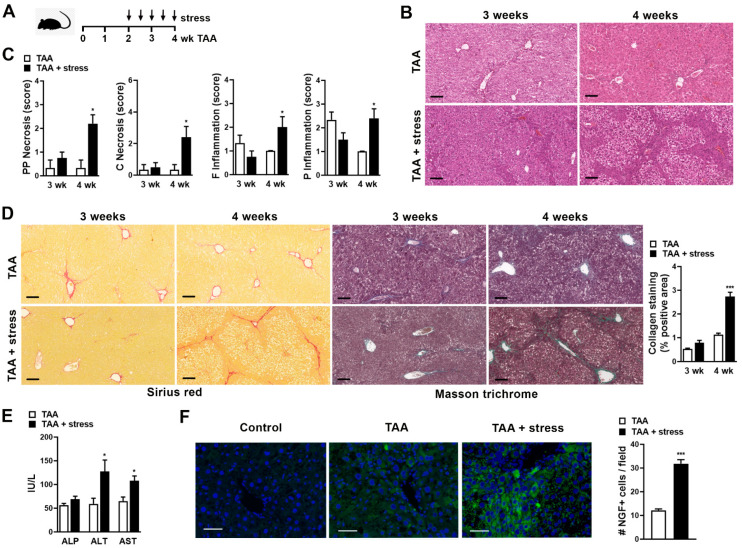
(**A**) Experimental murine model: Thioacetamide (TAA) was administered for 3 or 4 weeks (wk) to induce fibrosis and stress stimulus was performed by exposition to sound stress for the duration of 24 h starting on week 2 of TAA administration, twice a week. (**B**) Representative images of H&E staining at 3 and 4 weeks with fibrosis (TAA) or fibrosis with stress (TAA + stress). Bar = 100 µm. (**C**) Score of periportal and periseptal (PP) necrosis, confluent (C) necrosis, focal (F) inflammation, and portal (P) inflammation in livers at 3 or 4 weeks (wk) of fibrosis (TAA) or fibrosis with stress (TAA + stress). Results are expressed as mean score ± S.E.M. * *p* < 0.05 vs. TAA (Kruskal–Wallis test). (**D**) Representative images of Sirius red and Masson’s trichrome staining on liver sections. Bar = 100 µm. Quantification of collagen deposits based on Sirius red-stained sections was performed by morphometric analysis and % positive area ± S.E.M was depicted. *** *p* < 0.001 vs. TAA (Kruskal–Wallis test). (**E**) Alkaline phosphatase (ALP), aspartate transaminase (AST), and alanine aminotransferase (ALT) were measured in serum. * *p* < 0.05 vs. TAA (Mann–Whitney test). (**F**) Representative images and NGF expression in liver as number of NGF^+^ cells/field ± S.E.M is shown. Negative immunofluorescence control without NGF antibody is also shown (control). Bar = 50 µm. *** *p* < 0.001 vs. TAA (Mann–Whitney test).

In order to analyze whether stress might be detrimental on liver fibrosis, TAA was chronically administered in mice with or without stress exposition. Stress stimulus was performed twice a week by exposition to sound stress for the duration of 24 h starting on week 2 of TAA administration, and liver fibrosis was evaluated in mice without stress (TAA) and with stress stimulus (TAA + stress) after 3 or 4 weeks of TAA exposure (Figure 1A).

To evaluate the effect of stress on liver necroinflammatory activity, we performed histological analysis and Knodell histological activity index (HAI). At 3 weeks of TAA administration, focal and periportal inflammation was observed without significant difference between mice exposed or not to stress (Figure 1B,C, 3 weeks). Moreover, the HAI score was similar for both experimental groups (5 (3–6) vs. 5 (2–5)). However, at 4 weeks of TAA administration, stressed mice-hepatocytes showed degenerative changes with increased periportal and periseptal necrosis as well as necrosis in some or most of the perivenular areas. In addition, stressed mice presented increased focal parenchymal and portal inflammation compared to TAA mice (Figure 1B,C, 4 weeks). Thus, mice that were administered with TAA for 4 weeks and received a stress stimulus for 2 weeks presented a significantly higher HAI than mice that only were administered with TAA (10 (3–11) vs. 2 (2–4), respectively, *p* < 0.01). Analysis of Sirius red and Masson’s trichrome staining of liver sections showed more fibrous expansion of portal areas with a marked portal to portal as well as portal to central bridging in stressed mice at 4 weeks compared to mice not exposed to stress and to mice treated for 3 weeks with TAA with or without stress stimulus (Figure 1D). Next, we determined fibrosis stages using the Ishak score. Animals that were administered for 3 weeks with TAA with or without stress presented the same Ishak score (2 (2–3)). However, a difference in the score was observed at 4 weeks of TAA administration: Stressed mice showed a significantly increased scoring than TAA mice (2 (2–3) vs. 4 (4–5) in TAA vs. TAA + stress, respectively, *p* < 0.05). Collagen quantification on Sirius red staining revealed that one week of stress did not alter collagen deposition compared to TAA mice (3 weeks). In contrast, an increase in collagen was observed after 2 weeks of stress in comparison to TAA mice (4 weeks) (Figure 1D). Moreover, an increase in hepatic enzymes ALT and AST was observed in mice that were administered with TAA for 4 weeks and received a stress stimulus compared to the TAA group (Figure 1E). Due to the observation that 2 weeks of stress increased fibrosis in mice (4 weeks of TAA), we continued with this experimental setting henceforth.

To further understand the mechanism by which stress accelerates the TAA-induced fibrosis process, we then evaluated the expression of NGF in the liver, since this neurotrophin is a stress-mediator [16] and we have also previously found increased in mice exposed to stress [17]. In the TAA group, a low expression of NGF distributed in all the parenchyma was observed (Figure 1F, middle panel), however, stress exposure increased the expression of NGF, particularly in the fibrosis tracts (Figure 1F, right panel). This result shows in a model of chronic liver injury that stress challenge increased liver fibrosis by the up-regulation of NGF expression.

### 2.2. Blocking of NGF Pathways Disrupt Deleterious Effect of Stress in Experimental TAA-Mediated Fibrosis

**Figure 2:**
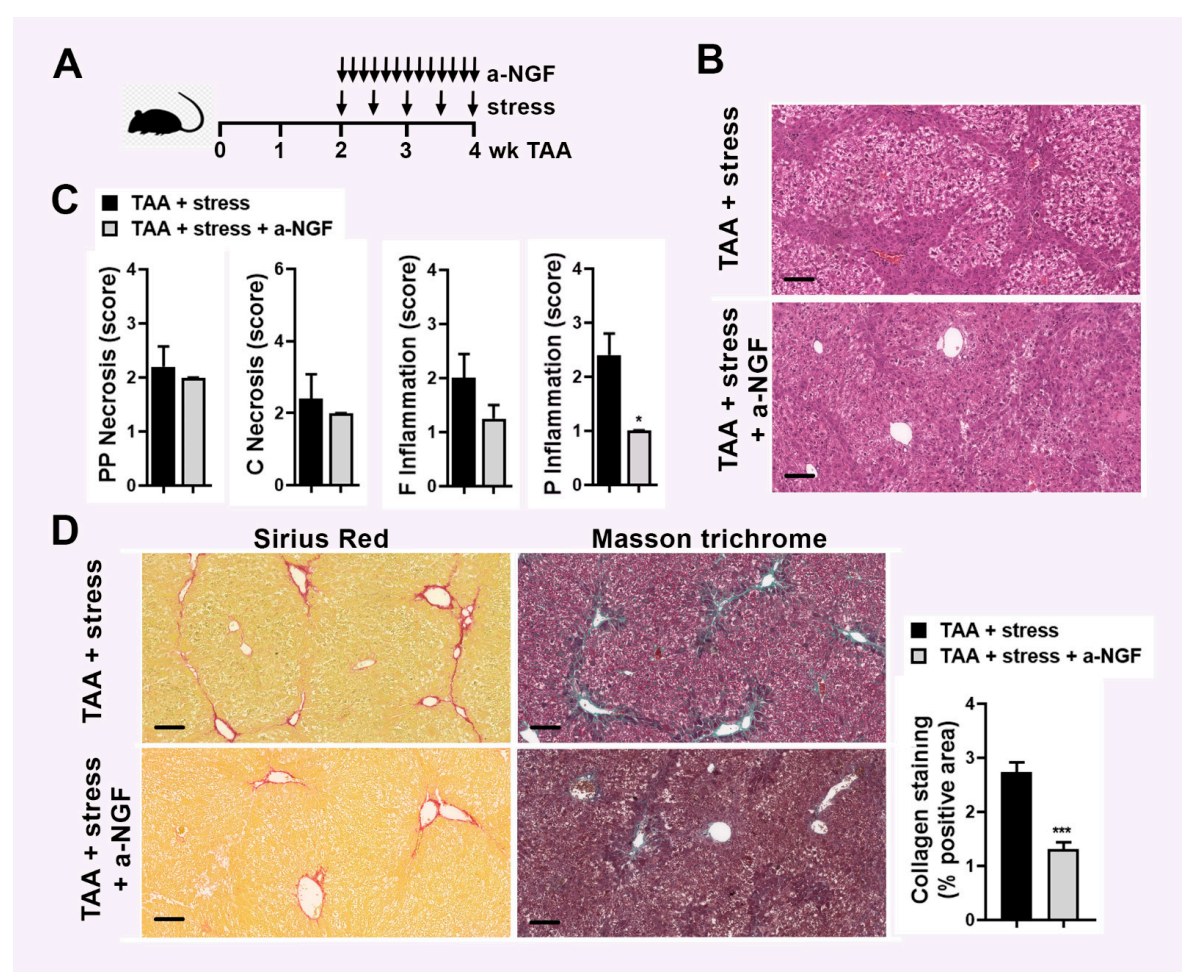
(**A**) Experimental murine model: Thioacetamide (TAA) was administered for 4 weeks (wk) to induce fibrosis, stress stimulus was performed by exposition to sound stress for the duration of 24 h starting on week 2 of TAA administration, twice a week, and for NGF blocking, mice were i.p. injected with neutralizing antiserum against NGF daily between week 2 and week 4. (**B**) Representative images of H&E staining on liver sections of mice with fibrosis and stress (TAA + stress) and injected with neutralizing antiserum against NGF (a-NFG). Bar = 100 µm. (**C**) Score of periportal and periseptal (PP) necrosis, confluent (C) necrosis, focal (F) inflammation, and portal (P) inflammation. Results are expressed as mean score ± S.E.M. * *p* < 0.05 (Kruskal–Wallis test). (**D**) Representative images of Sirius red and Masson’s trichrome staining on liver sections. Bar = 100 µm. Quantification of collagen deposits based on Sirius red-stained sections was performed by morphometric analysis and % positive area ± S.E.M was depicted. *** *p* < 0.001 (Kruskal–Wallis test).

After observing that NGF is upregulated in the liver of mice after TAA and stress challenge, we decided to neutralize it and evaluate the effect on liver fibrosis. For that purpose, the same experimental setting was performed: TAA administration for 4 weeks, stress stimulus twice a week starting on week 2, and for NGF blocking, mice were i.p. injected with neutralizing antiserum against NGF daily between week 2 and week 4 (Figure 2A). Histological analysis revealed that the blockage of NGF presented a lower HAI score than stressed mice (6 (6–7) vs. 10 (3–11), respectively, *p* < 0.05). Although no significant difference in piecemeal and confluent necrosis was observed, a significant decrease in the portal inflammation was detected (Figure 2B,C). Sirius red and Masson’s trichrome analysis revealed that in NGF-blocked mice, reduced portal to portal fibrous bridges and no portal to central vein bridges were detected (Figure 2D). In line with this, a decreased stage in the Ishak score was observed in mice that were administered with anti-NGF compared to stressed mice (3 (3–4) vs. 4 (4–5), respectively). Quantification of collagen deposition was performed and a decrease in the liver of mice that received the anti-NGF antibody compared to stressed mice was observed (Figure 2D). These results suggest that the deleterious effect of stress on fibrosis was partially reversed by blocking NGF.

### 2.3. NGF Administration during Experimental TAA Model Boosts Liver Fibrosis

**Figure 3:**
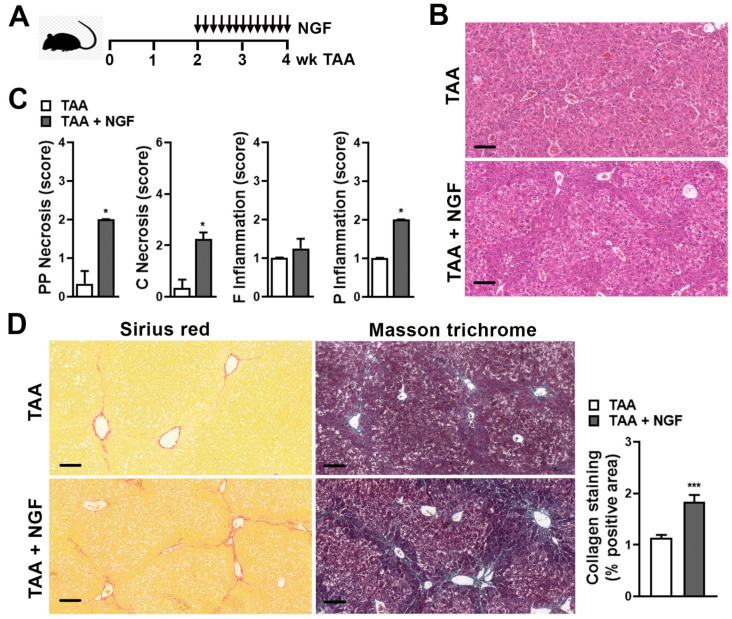
(**A**) Experimental murine model: Thioacetamide (TAA) was administered for 4 weeks (wk) to induce fibrosis and NGF administration was performed daily starting on week 2 of TAA administration. (**B**) Representative images of H&E staining on liver sections of mice with fibrosis (TAA) and fibrosis and NGF administration (TAA + NGF). Bar = 100 µm. (**C**) Score of periportal and periseptal (PP) necrosis, confluent (C) necrosis, focal (F) inflammation, and portal (P) inflammation. Results are expressed as mean score ± S.E.M. * *p* < 0.05 (Kruskal–Wallis test). (**D**) Representative images of Sirius red and Masson’s trichrome staining on liver sections. Bar = 100 µm. Quantification of collagen deposits based on Sirius red-stained sections was performed by morphometric analysis and % positive area ± S.E.M is shown. *** *p* < 0.001 (Kruskal–Wallis test).

The next aim was to evaluate if NGF treatment could mimic the effect on liver necrosis, inflammation, and fibrosis elicited by stress stimulus. For this purpose, mice administered with TAA were also injected with pro-NGF daily, starting at week 2 up to week 4 (Figure 3A). The level of hepatocellular damage was compared to mice without NGF administration. H&E staining demonstrated an increase in hepatocyte vacuolar degeneration around the central vein and periportal hepatocyte necrosis in the NGF-treated group (Figure 3B). In fact, an increase in the HAI score was observed in the NGF-treated group compared to the TAA group (8 (7–8) vs. 2 (2–4), respectively, *p* < 0.05). This increase in the HAI score was mainly due to the increase in periportal, perivenular necrosis, and portal inflammation but no significant difference in focal inflammation was observed (Figure 3B,C). In NGF treatment, an increase in the collagen bridges compared to the TAA group was observed (Figure 3D). The fibrosis Ishak score demonstrated a slight increase in NGF-treated animals compared to the TAA group (3 (2–3) vs. 2 (2–3), respectively). In addition, collagen quantification indicated that more deposition of collagen occurred in NGF-treated mice compared to TAA mice (Figure 3D). These results show that NGF treatment accelerates liver fibrosis to a similar extent than stress challenge.

**Figure 4:**
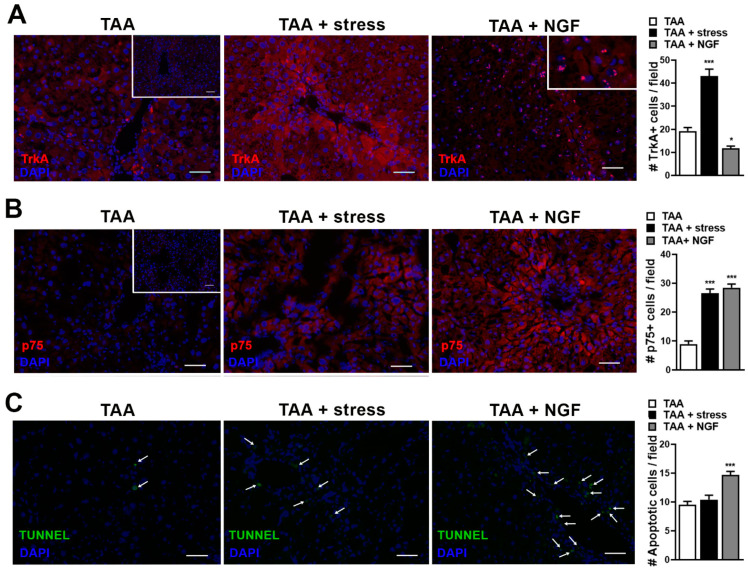
Representative images and number of TrkA^+^ cells/field ± S.E.M (**A**) and number of p75+ cells/field ± S.E.M (**B**) on liver sections of mice with fibrosis (TAA), fibrosis and stress (TAA + stress), and fibrosis and NGF administration (TAA + NGF). *** *p* < 0.001 and * *p* < 0.05 vs. TAA (Kruskal–Wallis test). Immunofluorescence negative controls without primary antibodies are shown as inset in left panels. (**C**) TUNEL staining (arrows) and a number of apoptotic cells/field ± S.E.M on the same experimental groups. *** *p* < 0.001 vs. TAA (Kruskal–Wallis test). Bar = 50 µm.

In order to better understand the role of NGF produced by stress stimulus on liver fibrosis, we evaluated the expression of the receptors involved in NGF responses, tropomyosin-receptor-kinase A (TrkA), and p75 neurotrophin receptor (p75NTR). We found an increase in both TrkA and p75 expression in stressed mice compared to TAA-treated mice (Figure 4A,B, left and middle panels). However, when the same analysis was performed in NGF-treated mice a decrease in TrkA and an increase in p75 expression compared to TAA group was observed (Figure 4A,B, left and right panels). Interestingly, in the NGF-treated mice, not only a decrease in TrkA expression, but also a nuclear localization of the receptor was observed (Figure 4A, insert in right panel). It has been previously described that depending on the expression level of both receptors, the effect of NGF binding has different outcomes. Binding of NGF to TrkA mediates survival and differentiation, and these effects have been found to be boosted upon the co-binding of TrkA and p75NTR [18]. On the other hand, several evidences demonstrated that p75NTR in the absence of TrkA triggers apoptosis [18]. For that reason, we evaluated apoptosis induction by TUNEL assay, revealing that only in the NGF-treated group, an increase in TUNEL positive cells was observed (Figure 4C). Interestingly, in NGF-treated mice both p75 increase and apoptosis induction were observed with a predominant localization around portal veins and fibrotic tracts (Figure 4B,C, right panels).

### 2.4. Altered Liver Glycosylation Accompanies the Progression of Stress-Induced Liver Fibrosis

In several pathological conditions, changes in the glycosylation pattern were observed. The study of glycosylation in liver diseases has been focused on the evaluation of serum proteins as a non-invasive method for diagnosis and prognosis, since most of glycosylated proteins present in serum are synthesized by the liver. It seems that even liver diseases of a different etiology share some alterations of glycosylation, such as an increase in fucosylation, branching, and bisecting N-acetylglucosamine [19]. However, few reports demonstrated the pattern of glycosylation in the liver tissue, and there are no reports describing the changes in glycosylation associated to stress.

**Figure 5:**
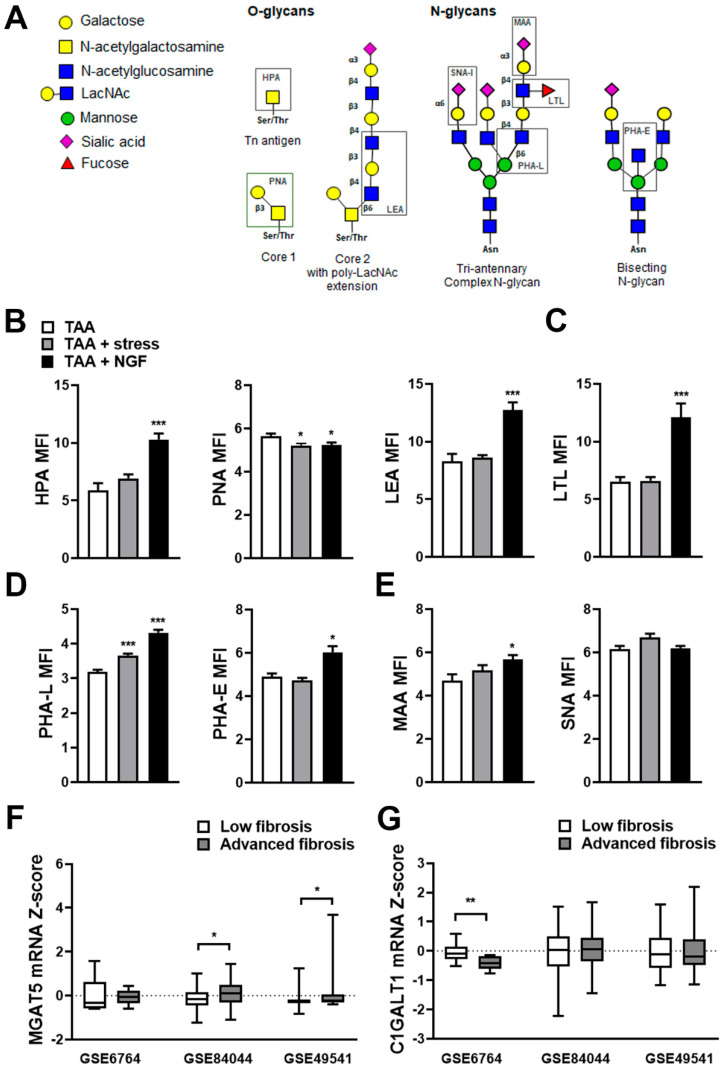
(**A**) For an analysis of the glycophenotype, lectins were used to detect different types of glycosylation. O-glycan structures were recognized by Helix pomatia agglutinin (HPA; Tn-antigen) and Arachis hypogaea lectin (PNA; core 1). Lycopersicon esculentum lectin (LEA) recognizes polyLAcNac sequences. Phaseolus vulgaris lectin (PHA-L) recognizes β1-6GlcNAc-branched complex N-glycans and Phaseolus vulgaris lectin E subunit (PHA-E) identifies glycans with a bisecting modification. Fucosylation was determined by Lotus tetragonolobus lectin (LTL) and finally sialyation was determined using the Maackia amurensis lectin (MAA) and Sambucus nigra agglutinin (SNA-I) which bind to α2,3- and α2,6-linked sialic acid, respectively. Quantification of O-glycan (**B**), fucosylation (**C**), N-glycosylation (**D**), or sialylated glycan (**E**) was performed on liver sections of mice with fibrosis (TAA), fibrosis and stress (TAA + stress), and fibrosis and NGF administration (TAA + NGF). Mean fluorescence intensity (MFI) ± S.E.M is shown. * *p* < 0.05 and *** *p* < 0.001 vs. TAA (Kruskal–Wallis test). Expression of MGAT5 (**F**) and C1GALT1 (**G**) in patients with fibrosis due to HCV (GSE6764), HBV (GSE84044), or NAFLD (GSE49541). * *p* < 0.05 and ** *p* < 0.01 (Mann–Whitney test).

To further analyze the effect of stress on fibrosis, we decided to explore the glycophenotype. To this aim, a panel of lectins that recognize specific glycans on a cell surface was used (Figure 5A). To determine the presence of O-glycan structures, the lectins *Helix pomatia agglutinin* (HPA; Tn-antigen) and *Arachis hypogaea* (PNA; core 1) were assayed. *Lycopersiconesculentum lectin* (LEA) recognizes polyLAcNac sequences on N- and O-glycans. To evaluate the sialylation, the lectins *Maackiaamurensis* (MAA) which binds to α2,3-linked sialic acid and *Sambucus nigra agglutinin* (SNA-I) that recognizes α2,6-linked sialic acid were used. We also tested the *Phaseolus vulgaris lectin L subunit* (PHA-L) which recognizes β1,6GlcNAc-branched complex N-glycans, *Phaseolus vulgaris lectin E subunit* (PHA-E) to identify N-glycans with a bisecting modification and *Lotus tetragonolobus lectin* (LTL), which has specificity toward α-linked L-fucose. We observed in both stressed mice and NGF-treated mice a downregulation of core 1 O-glycans (PNA) expression (Figure 5B, middle panel) and an increase in PHA-L binding glycans (Figure 5D, first panel). However, mice treated with NGF also presented increased expression of Tn antigen (HPA) and polyLAcNac sequences (LEA) (Figure 5B, left and right panel respectively). Moreover, increased expression of LTL (Figure 5C) and PHA-E (Figure 5D, right panel) reactive glycans as well as sialylated MAA-reactive glycans (Figure 5E, left panel) were observed also in the NGF-treated group. For SNA-I reactive glycans, a similar expression in the 3 experimental groups was observed (Figure 5E, right panel).

### 2.5. Differential Expression of Glycosylation Enzymes Is Observed in Fibrosis Progression in Patients

As we have found an increase in PHA-L binding and a decrease in PNA in both stressed and NFG-treated mice, enzymes responsible for these glycosylation patterns were evaluated in human samples. N-acetylglucosaminyltransferase V (MGAT5) is responsible for the production of β1,6 GlcNAc-branched complex N-glycans [20], and N-acetylgalactosamine 3β-galactosyltransferase 1 (C1GALT1) catalyzes the synthesis of the core 1 O-glycan structure, a precursor for many mucin-type O-glycans [21]. We analyzed the expression of both enzymes in 3 datasets in order to analyze their expression in patients with the three most common causes of liver fibrosis: HCV, HBV, or NAFLD. We observed a significantly upregulated expression of MGAT5 in HBV- and NAFLD-associated fibrosis in patients with advanced disease (advanced fibrosis) compared to those at the initial stage of the disease or without fibrosis (Figure 5F). For C1GALT1, the patients with advanced fibrosis due to HCV demonstrated a decrease in its expression compared to patients with low level of fibrosis and no significant differences were found in patients with fibrosis associated to HBV or NAFLD (Figure 5G).

## 3. Discussion

Several reports have partially demonstrated that stress contributes to the fibrosis induction and/or liver injury [22], however, this is the first report to identify the NGF as a critical mediator responsible for the deleterious effect of stress observed during experimental liver fibrosis. We observed increased NGF expression in the liver of stressed-mice and blockage of the NGF reduced fibrosis induced by a stress stimulus. Moreover, NGF-treated animals presented increased fibrosis similar to stressed animals. We conclude that NGF partially mimics the effect of stress on fibrosis progression. It has been previously reported that social and physical stressors induced the increase of the NGF both in the central nervous system and in the periphery [16]. Although NGF effects were initially described on neuronal cells, it has been demonstrated that the NGF also functions in other tissues [23]. Concerning the liver, the NGF has been found in rat and human HSCs both in normal liver and in CCl_4_- or HCV-induced cirrhosis [24]. An increase of NGF expression was also described in HSCs after 48 h of D-Galactosamine treatment in rats [24]. Our results clearly demonstrate that stress increases fibrosis and the NGF is involved in this process.

It is well established that around 90% of hepatocellular carcinoma arises in the context of chronic inflammation and from within a fibrotic liver [3]. Some experimental and epidemiologic studies indicated that psychological stress is associated with the initiation, progression, and dissemination of tumors [25,26]. Among the mechanisms involved, the dysregulation of the immune system is the most studied. In tumor patients, chronic stress has been found to influence the immune response at different stages such as suppression of the protective immunity, exacerbation of chronic inflammation, and enhancement of immunosuppression [27]. Stress also induces the secretion of different signaling molecules such as catecholamines and the adrenergic receptors are present in the brain, kidney, and liver, among other tissues. The activation of adrenergic signaling have been found in some tumors suggesting the role of catecholamines in tumor initiation and progression [28]. Although our studies did not focus on tumor initiation or development, the increased fibrosis observed after stress stimulus suggest that stress contributes to the chronic inflammation and acceleration of liver fibrosis, which subsequently imposes a risk factor for hepatocellular carcinoma.

NGF exerts its biological effects upon ligation to a high affinity receptor, TrkA, and to the low-affinity and non-selective p75NTR. The cellular response to NGF is determined by the combination of receptor expression: TrkA mediates survival and differentiation, and these effects of NGF have been found to be boosted upon the co-binding of TrkA and p75NTR. On the other hand, several evidences demonstrate that p75NTR in the absence of TrkA triggers apoptosis [18]. As observed with NGF, TrkA and p75NTR have been found in HSCs associated to fibrosis [24,29]. It was also demonstrated in a mouse model of fatty liver disease that NGF and p75NTR were upregulated [30,31]. Although TrkA and p75NTR are receptors, a cytoplasmic localization for both proteins and a nuclear localization for TrkA was previously observed in hepatocytes and activated hepatic stellate cells [32], similar to our findings. A study on liver biopsies from patients with HVB, HVC, and non-viral hepatitis, demonstrated that the level of p75 mRNA was increased proportionately to the degree of fibrosis, with significantly higher levels in livers in fibrosis stages 3 and 4 [33]. Our results, demonstrating that stress increased the expression of both TrkA and p75 and the fibrosis in the liver, are in line with previous data that have demonstrated that the expression of both receptors, TrkA and p75, were associated to the activation of HSCs observed in the progression of fibrosis [34]. However, in the NGF-treated mice, we observed a decrease in TrkA expression with an increase in apoptosis induction. In vitro experiments demonstrated that NGF induced apoptosis of HSCs [29], and in an acute CCl_4_ model of liver injury, NGF was expressed by hepatocytes associated to the induction of apoptosis of HSCs [35]. Moreover, Asai et al. demonstrated that after liver hepatectomy, NGF and p75NTR were increased and in vitro treatment of HSCs with NGF induced apoptosis [36]. Several data indicated that apoptosis of activated HSCs is involved in the regression of fibrosis [37] and regarding our results we can suppose that in NGF-treated mice the induction of apoptosis triggered by NGF through p75NTR could be associated with a slight decrease on fibrosis compared to stressed mice.

Over the last years, it has become more evident that protein glycosylation plays an important role in the pathogenesis and progression of several diseases. Glycosylation is a co- and post-translational modification that involves the endoplasmic reticulum and the Golgi apparatus. Most of the proteins expressed on the cell surface are glycosylated and the most common glycosylation include the addition of N-linked glycans to the amide nitrogen of asparagine (Asn) side chains and O-linked glycans to the hydroxyl groups of serine (Ser) and threonine (Thr) side chains [38,39]. Carbohydrates on glycoproteins are important for intra- and intercellular communication, such as the interaction between cells and with the extracellular matrix [40]. In liver disease, studies were focused on the pattern of glycosylation of serum proteins: An increase in fucosylation and branching of haptoglobin was observed in alcoholic liver disease [41]. Similarly, α1-antitrypsin, α1-acid glycoprotein, and haptoglobin were found hyperfucosylated in the serum of patients with bile-related liver disease [42]. Another report demonstrated increased fucosylation in α1-acid glycoprotein in the serum of patients with alcoholic liver disease, HVB, HVC, and cirrhosis [43]. However, data related to the glycophenotype in the liver tissue are scarce.

Several receptors on liver cells are glycosylated, and it has been described that not only the binding of some proteins to these receptors relies on carbohydrate moieties [19], but also the availability of the receptor on the cell surface depends on its glycosylation [44]. It has been previously demonstrated that β1,6GlcNAc-branched N-glycans stabilize TrkA on the cell surface and allow ligand interaction [20]. The biosynthesis of these N-linked oligosaccharides depends on the activity of N-acetylglucosaminyltransferase V (Mgat-5) [45], and it has been demonstrated that Mgat5-modified N-glycans on transforming growth factor β (TGFβ) receptors delayed their removal from cell surface [46]. Moreover, in a mouse model of renal fibrosis, glucosamine hydrochloride supplementation inhibited a pro-fibrotic pathway by decreasing N-glycosylation of TGFβ receptor type 2 (TGFR2), thus inhibiting its translocation to the cell surface membrane [47]. Considering that TGFβ is a known mediator of fibrosis and our results showed that NGF is involved in fibrosis development, we hypothesize that the increase in branched complex N-glycans observed in the liver of stressed and NGF-treated mice allows both receptors to be on cell surface and consequently interact with their ligands. We also demonstrated that in the liver from patients with different liver diseases, fibrosis progression correlates with the increase in Mgat-5 expression. These results are in line with data obtained in the mouse model and suggest that similar glycodeviations are observed also in humans during liver fibrosis progression.

Psychological stress is increasingly frequent in current society; thus, a better understanding of this process could be beneficial for the clinical management of patients with liver disease. Our results clearly demonstrated the effect of stress on the initial stages of fibrosis, and the impact of NGF on increasing liver injury. On the other hand, this study showed that stress and particularly the NGF, induced several glycophenotype modifications on cell surface and a correlation with human data were also observed. For that reason, evaluating the glycophenotype pattern in patients with liver disease could contribute to fibrosis staging and eventually to develop new diagnostic strategies.

## 4. Materials and Methods

### 4.1. Liver Fibrosis Model and Animal Experiments

Seven- to eight-week C57BL/6 male mice were purchased from Charles River (Germany) and maintained in our animal facility with a 12L/12D cycle with free access to food and water. Mice were injected intraperitoneally (i.p.) with thioacetamide (TAA, Sigma Aldrich, Germany, 0.2 mg/g body weight) three times a week for 4 weeks [48]. Mice were exposed to sound stress for the duration of 24 h, starting on week 2, twice a week for 3 weeks [49]. For NGF treatment, on week 2, mice from the control or TAA group were i.p. injected with murine pro-NGF (7S, 20 μg/mouse per day, Sigma–Aldrich, St. Louis, MO, USA) daily. For NGF neutralization, mice were i.p. injected with 200 μL of non-immune rabbit serum (3.2 μg/kg, Sigma–Aldrich, St. Louis, MO, USA) or with a neutralizing antiserum against NGF (3.2 μg/kg, Sigma–Aldrich, St. Louis, MO, USA) daily between week 2 and week 4 as described previously [50]. At least 5 animals per experimental group was analyzed. Mice were sacrificed on day 30, and blood and liver tissue were obtained. Small pieces of each lobe were snap frozen in liquid nitrogen, embedded in optimal cutting temperature compound, and stored at −80 °C until analysis, or fixed in 10% phosphate saline-buffered neutral formalin.

### 4.2. Histology and Liver Fibrosis Analysis

Paraffin embedded liver samples were cut in 5-µm thick sections and stained with haematoxylin-eosin (H&E), Masson’s trichrome, and Sirius red according to standard procedures. Liver necroinflammation was graded per the Ishak-modified Knodell Histological Activity Index (HAI), analyzing periportal and periseptal necrosis (score 0–4), confluent necrosis (score 0–6), focal inflammation (score 0–4), and portal inflammation (score 0–4). Fibrosis was assessed according to the scoring system proposed by Ishak et al. (no fibrosis 0 and cirrhosis 6) [51]. Analyses were performed in a blinded fashion and data presented as median and range. Quantitative analysis of collagen content was performed by computerized morphometric analysis on samples stained with Sirius red and digitally scanned by a high-resolution bright field and fluorescence slide scanner (Pannoramic MIDI BF/FL, 3DHISTECH Ltd., Budapest, Hungary). For this purpose, light microscope images (200X), excepting large centrilobular veins and large portal tracts were analyzed using Pannoramic Viewer 1.15.4 (3DHISTECH Ltd.). Values were expressed as a percentage of the positive area [52].

### 4.3. Liver Enzymes

Enzyme levels of aspartate aminotransferase (AST), alanineaminotransferase (ALT), and alkaline phosphatase (ALP) were determined as previously [48].

### 4.4. Immunofluorescence

Eight micrometer cryosections were blocked and incubated with primary antibodies: Anti-TrkA (Santa Cruz Biotechnology; sc-118) or anti-p75 (Santa Cruz Biotechnology; sc-5634). Rhodamine-labeled secondary antibodies (Jackson Immuno-Research 111-026-045) were used followed by 4´,6-diamidino-2-phenylindole (DAPI). Paraffin-embedded liver samples were also incubated with anti-NGF (Santa Cruz Biotechnology; sc-549 clone M-20). FITC-labeled secondary (Jackson Immuno-Research 115-095-047) were used followed by DAPI. Immunofluorescence controls without the primary antibody were also performed. Sections were analyzed using an immunofluorescence microscope (Axio Imager.M2, Carl Zeiss, Oberkochen, Germany). 

### 4.5. Terminal dUTP Nick-End Labeling (TUNEL) Staining

To evaluate apoptotic cells in liver cryosections, we used our standard TUNEL staining protocol as previously described [53].

### 4.6. Glycophenotype Analysis

Cryosections of liver were prepared at 8 μm. Briefly, slides were washed in TBS and blocked with the Biotin Blocking system (X0590, DAKO Corporation, Hamburg, Germany) for 20 min in a humid chamber at RT. Afterwards, slides were blocked with Carbo-Free Blocking Solution (SP-5040, Vector Laboratories) for 30 min in a humid chamber at RT. Subsequently, slides were incubated with biotinylated lectins binding a particular glycan structure (EY Laboratories, CA, USA) diluted in Carbo-Free Blocking Solution for 16 h at 4 °C in a humid chamber: Sambucus nigra agglutinin, SNA-I (10 ng/mL; BA-6802-1), Phaseolus vulgaris lectin, PHA-L (20 ng/mL; BA-1801-2), Helix pomatia agglutinin, HPA (20 ng/mL; BA-3601-1), or Phaseolus Vulgaris Erythroagglutinin, PHA-E (20 ng/mL; B-1125). Lectin-stained sections were then incubated with 2 μg/mL Streptavidin-Tetramethylrhodamine (S-870; Invitrogen) for 1 h in a humid chamber at RT. Subsequently, slides were incubated with FITC-labeled lectin (EY Laboratories) diluted in Carbo-Free Blocking Solution for 2 h at RT (Maackiaamurensis lectin, MAA (20 ng/mL; F-7801-2), Arachis hypogaea lectin, PNA (20 ng/mL; F-2301-1), Lycopersiconesculentum lectin, LEA (20 ng/mL; F-7001-1), or Lotus Tetragonolobus lectin, LTL (20 ng/mL; FL-1321)). Nuclei were counterstained with 4′,6-diamidino-2-phenylindole (DAPI) for 5 min at RT and mounted in Prolong Gold (P36930, Invitrogen, Germany). Liver sections were digitally scanned by a high-resolution bright field and fluorescence slide scanner (Pannoramic MIDI BF/FL, 3DHISTECH Ltd.), and staining was evaluated on virtual slides using Pannoramic Viewer 1.15.4 (3DHISTECH Ltd.).

### 4.7. Bioinformatic Analysis

The following databases from patients with fibrosis were downloaded from the Gene Expression Omnibus (GEO): GSE6764 patients with the hepatitis C virus (HCV) infection [54], GSE84044 patients with the hepatitis B virus (HBV) [55], and GSE49541 patients with nonalcoholic fatty liver disease (NAFLD) [56]. For all the datasets, the patients were assigned into two groups according to the stage of fibrosis, low (F0/F1) or advanced (F3/F4). Two probes were evaluated for MGAT5 (206720_at, 212098_at) and 3 probes for C1GALT1 (219439_at, 226105_at, 226107_at), and Z-scores were calculated to compare mRNA expression.

### 4.8. Statistics

Data are expressed as mean ± S.E.M. Statistical analysis was performed by the Mann–Whitney or Kruskal–Wallis test and differences were considered significant when *p* < 0.05.

## Data Availability

The data that support the findings of this study are available from the corresponding author upon reasonable request.
